# Epidermoid Cyst of the Clitoris: A Rare Cause of Clitoromegaly

**Published:** 2013-11-24

**Authors:** Manoj Saha

**Affiliations:** Department of Pediatric Surgery, Gauhati Medical College, Guwahati, INDIA.

**Dear Sir,**

Epidermoid cysts are slowly growing tumors that arise due to invagination of epidermis into dermis either spontaneously or following trauma. While common locations are the face, scalp, neck and trunk, external genitalia can also be affected with scrotal, labial or clitoral involvement. Clitoral location of this lesion may produce local sign of virilization in the form of clitoromegaly. This can cause significant parental worry and psychological disturbances in an older child. We report a girl with clitoromegaly due to epidermoid cyst.

A 5-year-old girl presented with a painless swelling at external genitalia noticed since early infancy. Swelling gradually increased in size. There was nothing significant in antenatal and postnatal history. Genital examination revealed a firm cystic swelling arising from the clitoris. It was not tender, mobile from side to side and firmly attached to the base of the clitoris. Mass was covered by normal skin antero-laterally and with mucosa on the dorsum. Urethral and vaginal openings were normal (Fig. 1). No signs of virilization were noted. Ultrasonography of the abdomen revealed normal ovaries and uterus. At operation, the cyst was excised through an inverted V shaped incision preserving the dorsal neurovascular bundles (Fig. 2). Clitoral reconstruction was done. Wound healed satisfactorily. Gross appearance and histopathology of the specimen were confirmatory of epidermoid cyst. At 2 years follow up her external genitalia appeared normal with preserved clitoris (Fig. 3).

**Figure:**
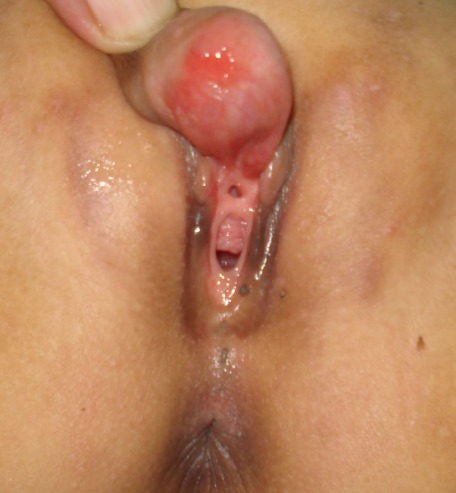
Figure 1: Extent of the cyst with normal urethral and vaginal openings

**Figure:**
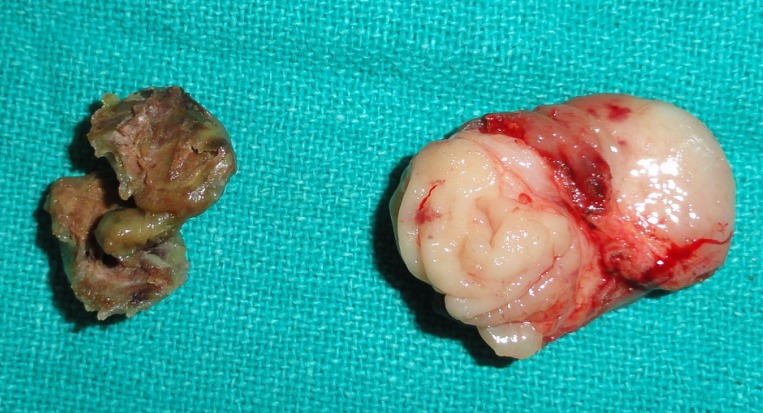
Figure 2: Excised specimen of the cyst with contents

**Figure:**
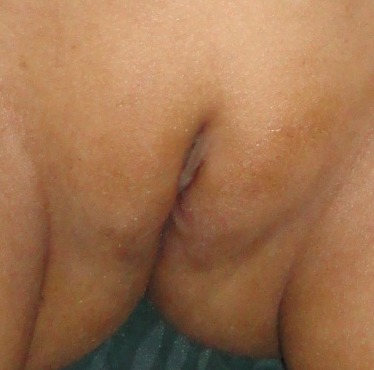
Figure 3: Appearance of clitoris at postoperative follow up

Causes of clitoromegaly can be hormonal, non-hormonal, pseudo-clitoromegaly, and idiopathic.[1] Endocrinopathies include: congenital adrenal hyperplasia(CAH) or adrenogenital syndrome, bilateral hilus cell tumor of the ovary, steroid producing gonadal tumors, adrenal androgen secreting carcinoma, Leydig cell tumor of the ovaries and metastatic carcino-sarcoma of the urinary bladder.[1-4] Common non-hormonal causes include neurofibromatosis (NF), epidermoid cyst, various syndromes and nevus lipomatous cutaneous superficialis etc.[4] A soft, mobile, non-tender mass in the clitoral region with absence of virilization is the typical physical finding, which is almost similar to ours. A detailed history and careful clinical examination can differentiate it from most of the other causes of clitoromegaly and spare the child from unnecessary investigations and can avoid delay in surgery.

Objectives of the treatment are straight-forward excision of the cyst with preservation of sexual arousal function and sensation, and cosmetic appearance. This can be achieved by preservation of the neurovascular bundles during dissection.[1] An inverted V shaped incision, as described by Celik et al, preserves neurovascular bundles and clitoral skin for cosmetic reconstruction of the clitoris.[5] Same was applied in index case.

## Footnotes

**Source of Support:** Nil

**Conflict of Interest:** None declared

